# Hypercalcemia, Staghorn Calculus and Xanthogranulomatous Pyelonephritis: A Diagnostic Challenge Unmasking Metastatic Urothelial Carcinoma

**DOI:** 10.7759/cureus.108612

**Published:** 2026-05-10

**Authors:** Jeanne Li Wen Ng, Irene Yuen Lin Yii, Nikhel Sachdev

**Affiliations:** 1 Internal Medicine, Singapore General Hospital, Singapore, SGP; 2 Endocrinology, Singapore General Hospital, Singapore, SGP

**Keywords:** hypercalcemia of malignancy, parathyroid hormone-related peptide (pthrp), staghorn calculus, urothelial carcinoma, xanthogranulomatous pyelonephritis

## Abstract

Xanthogranulomatous pyelonephritis (XGP) is a rare, chronic inflammatory renal condition often associated with urinary tract obstruction, nephrolithiasis, and recurrent infection. Diagnostically, it can mimic malignancy on both clinical presentation and imaging.

A 65-year-old woman presented with a three-week history of abdominal pain, fever, and giddiness. Cross-sectional imaging demonstrated a left staghorn calculus with hydronephrosis and features consistent with XGP and pyonephrosis. She underwent urgent percutaneous nephrostomy (PCN) with drainage of purulent material. During admission, she developed acute confusion and was found to have severe hypercalcemia (albumin-corrected calcium 3.7 mmol/L) with suppressed parathyroid hormone (PTH) and elevated parathyroid hormone-related protein (PTHrP). Positron emission tomography (PET)-CT imaging revealed widespread fluorodeoxyglucose (FDG)-avid lesions involving the left kidney, para-aortic lymph nodes, lungs, myocardium, and rectum. These findings were collectively worrisome for either disseminated infection or underlying malignancy. Tissue biopsy of a para-aortic lymph node demonstrated poorly differentiated metastatic carcinoma with squamous differentiation, ultimately establishing the presence of metastatic urothelial carcinoma, with involvement of the left kidney, lymph nodes, and lungs. The patient was referred for palliative systemic chemotherapy.

This case illustrates the complex interplay of multiple pathologies: Staghorn calculi not only leads to complications such as XGP and obstructive pyonephrosis, but is also associated with urothelial carcinoma. Through production of PTHrP, humoral hypercalcemia of malignancy further propagates nephrolithiasis. The case highlights the diagnostic challenge of distinguishing XGP and its infective complications from urological malignancy, and underscores the critical role of PTHrP in prompting evaluation for occult malignancy. Maintaining diagnostic vigilance is crucial, given the possible co-existence of multiple pathologies.

## Introduction

Xanthogranulomatous pyelonephritis (XGP) is a severe chronic inflammatory renal condition characterized by destructive granulomatous inflammation, often associated with long-standing urinary tract obstruction, nephrolithiasis and recurrent infection [1]. It is uncommon, accounting for up to 1% of cases of pyelonephritis [1]. It may present insidiously with non-specific symptoms such as abdominal pain, fever, and malaise. Radiologically and clinically, XGP is well known to mimic renal malignancy, posing a diagnostic challenge [1,2].

Parathyroid hormone (PTH)-independent hypercalcemia, particularly when mediated by parathyroid hormone-related protein (PTHrP), is most commonly associated with underlying malignancy, including urothelial carcinomas [3]. Co-existing infection and malignancy may obscure the clinical picture, delay diagnosis, and complicate management.

We report a diagnostically challenging case of XGP initially presenting with sepsis and obstructive uropathy, complicated by severe PTH-independent hypercalcemia, which ultimately led to the diagnosis of metastatic urothelial carcinoma. This case is unusual, specifically in its co-existence of multiple pathologies - XGP, staghorn calculus, metastatic cancer, and severe hypercalcemia - underscoring the importance of maintaining a high index of suspicion for occult malignancy in atypical presentations of hypercalcemia.

The case report was conducted in compliance with the Act on the Protection of Personal Information by fully anonymizing patient data. Written informed consent for publication was obtained from the patient. As this is a single case report, approval from an ethics committee was not required.

## Case presentation

A 65-year-old woman presented to the emergency department with three weeks of abdominal pain, giddiness, and tactile fever. The patient had a history of hypertension, hyperlipidemia, and bilateral chronic venous insufficiency, but had defaulted medications and follow-up for at least eight years. The abdominal pain was initially vague and generalized, with no associated dysuria or hematuria. On examination, she had stable vital signs with a low-grade fever. Generalized mild abdominal tenderness was present, with no guarding, rigidity or rebound tenderness. Bilateral renal punch was negative. 

Initial investigations done showed evidence of acute kidney injury, anemia, thrombocytosis, and raised infective markers (Table 1). Blood and urine cultures were sent. IV ceftriaxone and IV metronidazole were commenced to cover for intra-abdominal infection.

**Table 1:** Pertinent laboratory results Hb: Hemoglobin; WBC: White blood cells; PLT: Platelets; Cr: Creatinine; eGFR: Estimated glomerular filtration rate; CRP: C-reactive protein; Procal: Procalcitonin; cCa: Calcium corrected for hypoalbuminemia

Day of admission		1	3	5	7	9	11
Parameter	Reference Range						
Hb (g/dL)	12.0-16.0	7.6	-	7.8	-	-	7.9
WBC (x 10^9^/L)	4.0-10.0	14.0	-	15.9	-	-	10.8
PLT (x10^9^/L)	140-440	844	-	744	-	-	568
Cr (umol/L)	37-75	124	-	67	-	-	-
eGFR (mL/min/1.73m^2^)	-	39	-	82	-	-	-
CRP (mg/L)	0.2-9.1	255	306	-	-	-	130
Procal (ug/L)	<0.50	1.1	-	-	-	-	0.4
cCa (mmol/L)	2.09-2.46	-	3.7	3.0	2.8	2.3	2.2

In view of worsening abdominal pain, rising inflammatory markers, and abdominal X-ray findings of possible staghorn calculus (Figure 1), a CT scan of the abdomen and pelvis was arranged on day 3 of admission. This showed radiological findings consistent with XGP, possible pyonephrosis, with a large 3.6 cm staghorn calculus and associated hydronephrosis and perinephric fat stranding (Figure 2).

**Figure 1:**
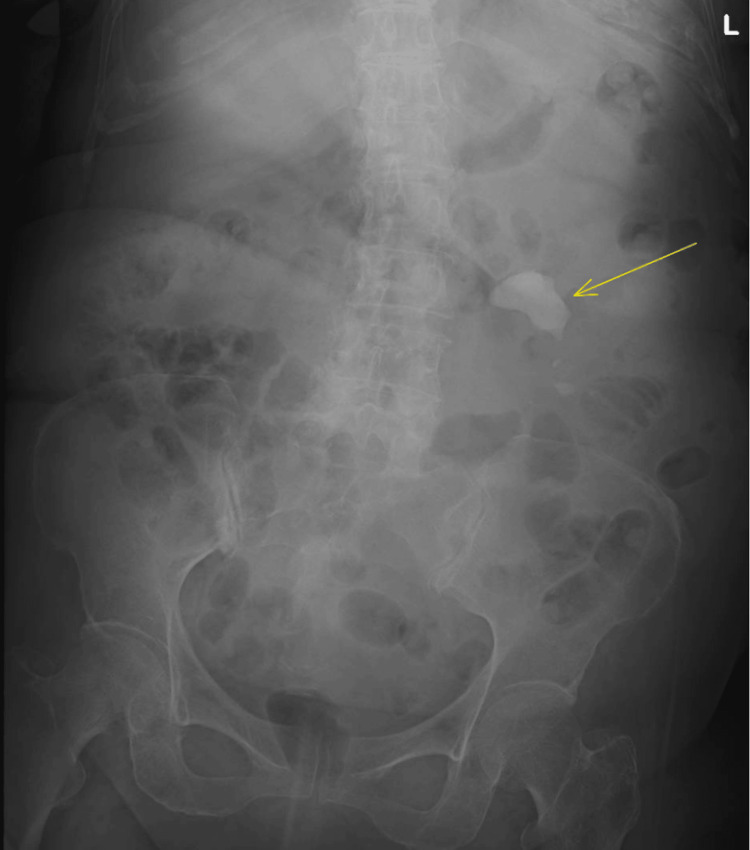
Supine abdominal X-ray showing coarse calcification projected over the left flank (arrow), raising suspicion for staghorn calculus

**Figure 2:**
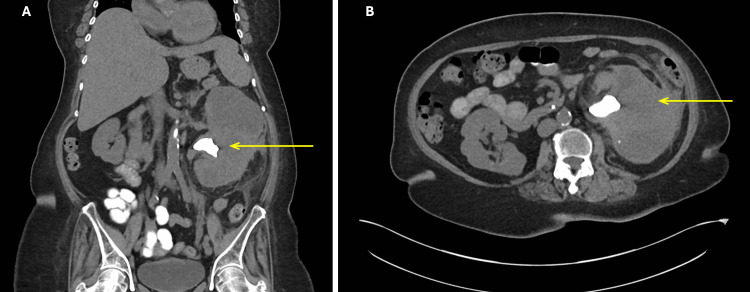
Cross-sectional CT images (A: sagittal, B: coronal) illustrating large 3.6 cm staghorn calculus (arrowed in A) with resultant XGP (bear paw sign - arrowed in B), with associated left hydronephrosis and markedly dilated calyces XGP: Xanthogranulomatous pyelonephritis

She subsequently spiked a fever and developed intense pain over the left flank. Urology was consulted and an urgent CT-guided left percutaneous nephrostomy (PCN) was performed, with 300 ml of pus drained during the procedure. The pus was sent for gram stain and culture, acid-fast bacilli (AFB) smear and culture, and fungal microscopy and culture - all of which returned negative, likely contributed by pre-treatment with antibiotics.

The patient then became agitated and confused, attempting to remove her IV cannula and climb out of bed. This was initially attributed to delirium from sepsis and pain. An extended electrolyte panel was sent, which revealed markedly elevated albumin-corrected calcium of 3.7 mmol/L. Phosphate was within normal limits and PTH (intact) (iPTH) was suppressed at 0.5 pmol/L.

The working diagnosis was that of symptomatic PTH-independent severe hypercalcemia complicated by confusion, nephrolithiasis and abdominal pain. She denied taking any dietary supplements. Aggressive IV hydration with IV normal saline 0.9% 3 L/day was initiated. She was simultaneously started on subcutaneous (SC) calcitonin 6 u/kg Q8H for 72 hours and given a dose of IV zoledronic acid 4 mg. Work-up for the underlying cause of hypercalcemia followed, summarized in Table 2. In particular, work-up was significant for an elevated PTHrP; myeloma panel returned negative, calcitriol levels were normal, and a contrasted CT scan of the thorax, abdomen, and pelvis revealed no evidence of malignancy.

**Table 2:** Work-up for underlying cause of hypercalcemia iPTH: Parathyroid hormone (intact); PO4: Phosphate inorganic; 25(OH)D3: 25-hydroxycholecalciferol; PTHrP: Parathyroid hormone-related protein; CT TAP: CT scan of the thorax, abdomen, and pelvis; NR: Normal range

Investigation	Result
iPTH (pmol/L)	0.5 (NR 0.9–6.2)
PO4 (mmol/L)	1.35 (NR 0.94–1.50)
25(OH)D3 (ng/mL)	17.4
Myeloma panel	Negative
PTHrP	26 pmol/L (NR <4.2)
Calcitriol	35 pg/mL (NR 18–78)
CT TAP	Negative for malignancy

Calcium levels down-trended gradually and normalized by day 9 of admission, along with a normalized renal function. Her abdominal pain resolved, and confusion did not recur. She completed two weeks of IV ceftriaxone, and a course of per oral cefuroxime was continued until outpatient review. She was discharged well with a left PCN in situ, with plans to follow up with Urology for discussion of elective left nephrectomy, and Infectious Diseases to review symptoms and trend infective markers. A positron emission tomography (PET)-CT scan was scheduled outpatient to further interrogate for presence of malignancy, given the unexplained hypercalcemia and the raised PTHrP.

The PET-CT scan was performed three weeks after discharge. This revealed widespread fluorodeoxyglucose (FDG)-avidity involving the hydronephrotic left kidney, para-aortic lymph nodes, lung nodules, basal myocardial ventricular septum, and a focus at the rectum. These findings were collectively worrisome for either disseminated infection or underlying malignancy, which prompted her re-admission from clinic for further evaluation and management. Hypercalcemia was also noted to have recurred, though it was asymptomatic. This was managed in her second admission with IV hydration, with good effect.

Thorough work-up for the multiple sites of FDG-avidity ensued, as summarized in Table 3.

**Table 3:** Work-up for multiple areas of FDG-avidity FDG: Fluorodeoxyglucose; PET: Positron emission tomography; ESBL: Extended-spectrum beta-lactamase; XGP: Xanthogranulomatous pyelonephritis; PCN: Percutaneous nephrostomy; AFB: Acid-fast bacilli; EGD: Esophago-gastro-duodenoscopy

PET-CT findings	Investigations and Management
Renal: Hydronephrotic left kidney with FDG uptake, perinephric fat stranding and a large staghorn calculus	Urine cultures grew ESBL producing *Klebsiella pneumoniae*. Antibiotics were escalated to IV meropenem accordingly. Discussion with nuclear medicine concluded that radiological findings of the left kidney favoured XGP over malignancy. Urology advised to consider alternative sites of biopsy as upfront biopsy of left kidney or left nephrectomy would come with significant risks. Urine from the left PCN was sent for cytology: acute inflammatory yield, no malignant cells.
Respiratory: multiple bilateral lung nodules showing FDG-avidity	Respiratory Medicine advised bronchoscopy with bronchoalveolar lavage would likely be of low yield given peripheral nature of nodules. Suggested discussion with Interventional Radiology for biopsy instead.
Lymph nodes: Enlarged and FDG-avid left para-aortic nodes	Interventional Radiology performed a CT-guided biopsy of the left para-aortic lymph node. Gram stain and culture, fungal microscopy, and AFB smear all returned negative. Histopathology report: Poorly differentiated carcinoma, consistent with metastatic carcinoma.
Cardiac: FDG-avid uptake noted at the basal myocardial ventricular septum	Transthoracic echocardiogram: Left ventricular ejection fraction of 60-65%, with no regional wall motion abnormality; no thrombus or vegetation. Cardiology consulted; FDG-avidity deemed non-specific, no follow-up required.
Gastrointestinal: A focus of FDG uptake seen at the rectum	Gastroenterology referred for endoscopic evaluation. EGD: Fundal gland polyp, otherwise normal. Colonoscopy: Sessile polyp in rectum, diverticular disease, hemorrhoids.

A multi-disciplinary discussion involving Urology, Respiratory Medicine, and Interventional Radiology concluded that the FDG-avid left para-aortic lymph node would be of highest yield for biopsy. A CT-guided biopsy of this lymph node was thus performed (Figure 3).

**Figure 3:**
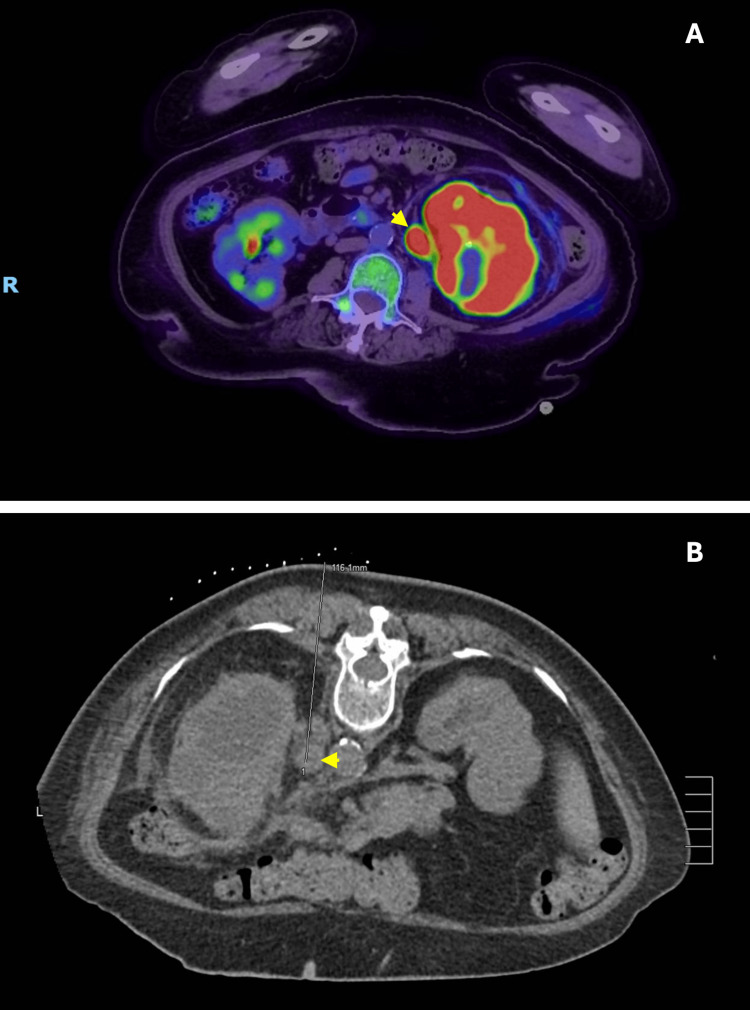
A: PET-CT image depicting intense FDG-avidity of the left kidney and neighboring left para-aortic lymph node (arrow); B: CT-guided biopsy of left para-aortic lymph node (arrow) PET: Positron emission tomography; FDG: Fluorodeoxyglucose

Histopathology for the left para-aortic lymph-node biopsy returned with findings consistent with poorly differentiated metastatic carcinoma (Figure 4). The morphologic features suggested a tumor with squamous differentiation, the most likely diagnosis being metastatic urothelial tract carcinoma given the co-positivity for p40 and GATA3. 

**Figure 4:**
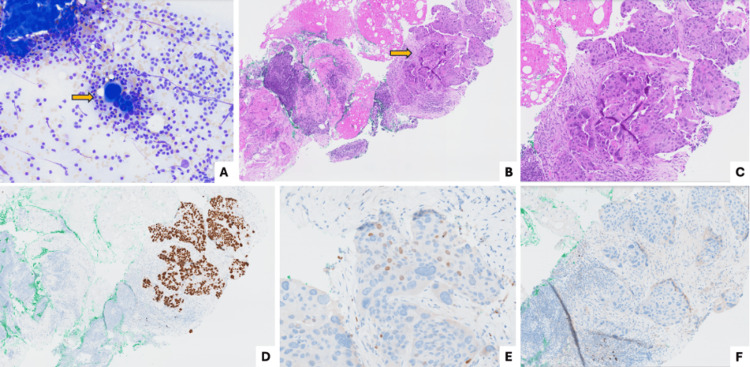
Left para-aortic lymph-node biopsy histopathology images A: Biopsy imprint showing small clusters of malignant cells in a lymphoid background (arrowed); B: H&E stain; and C: Magnified view of H&E stain. This shows round nests of tumour cells without any differentiation (arrowed) on a background of fragments of blood and nodal parenchyma; D: Diffuse and strong positivity for p40; E: Patchy weak positivity for GATA3; F: Negativity for CK20 H&E: Hematoxylin and eosin

The much less likely differential based on histopathology immunohistochemistry was that of squamous cell carcinoma (SCC), for which other anatomic sites of origin were also evaluated for appropriately. An MRI of the paranasal sinuses and a nasoendoscopy was performed and showed no evidence of head and neck SCC. There were also no respiratory symptoms reported and no dominant lung mass suggestive of lung SCC. There were no gynecological symptoms such as post-menstrual bleeding and, moreover, no concerning CT or PET scan findings in the adnexa region to suggest a cervical SCC.

The final diagnosis was hence that of metastatic urothelial carcinoma with involvement of the right kidney, lymph nodes and lung, given that the histopathology immunohistochemistry results were strongly in favor of this, and there were no clinical or radiological features to suggest the much less likely differential of SCC. Urology advised that a direct biopsy of the renal lesion would come with significant risks of bleeding, without much additional diagnostic clarity, given the features highly consistent with urothelial carcinoma. The patient herself was not keen to pursue a left nephrectomy. In support of this, the urologists opined that nephrectomy would not likely lead to meaningful survival benefit in light of her underlying metastatic cancer. The patient was referred to Medical Oncology and counseled for palliative systemic chemotherapy, with a prognosis given of likely less than one year. 

## Discussion

This case illustrates a cascade of events, revealing the complex interplay of multiple pathologies, summarized in Figure 5.

**Figure 5:**
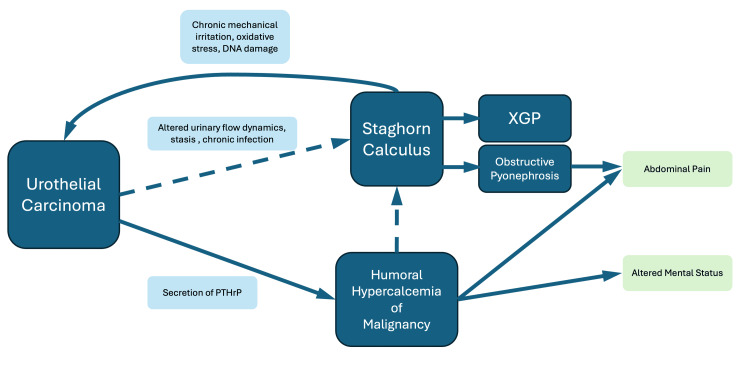
Schematic representation of the complex interplay of pathologies affecting the patient Dashed arrows indicate possible causation. Light blue boxes: Pathophysiology; Green boxes: Symptomatology Image created by authors using Microsoft PowerPoint.

The patient’s initial presentation was vague with a low-grade fever, generalized subacute abdominal pain, and giddiness. A broad list of differential diagnoses was considered at this stage, with an infective cause suspected foremost, in view of her markedly raised infective markers. Her symptoms evolved with the intensifying of abdominal pain, localization to the right flank, and high spiking fever. The constellation of symptoms also widened with the new development of altered mental status. In hindsight, her symptomatology was entirely consistent with the known complications of severe hypercalcemia - renal stones, abdominal groans, and psychiatric moans.

The extensive evaluation that followed, which included PET-CT, bidirectional endoscopy and imaging-guided biopsy, was driven by the lack of a readily identifiable cause for hypercalcemia and her raised PTHrP. The role of PTHrP in the diagnostic flow is elucidated here - specifically, that it proves most useful when the diagnosis is uncertain in cases of PTH-independent hypercalcemia. The positive PTHrP was a vital consideration in ascertaining the necessity and urgency of further evaluation. It increased the pre-test probability of an underlying malignancy, which impacted on the decision to arrange PET-CT imaging and pursue lymph-node biopsy in the acute setting. Had PTHrP returned negative, an alternative clinical course may have been considered, that is, to presume the FDG-avid lymph nodes were infection-related and to arrange interval imaging after an extended course of antibiotics. The potential diagnostic pitfall would have been to attribute all findings to chronic stone disease complicated by systemic infection, thereby missing the underlying malignancy. 

PTHrP is typically associated with SCCs of the lung, head/neck, and urothelial carcinomas [3]. The mechanism of hypercalcemia in urothelial carcinoma is predominantly mediated by tumor secretion of PTHrP causing humoral hypercalcemia of malignancy, rather than osteolytic hypercalcemia, and cases are often diagnosed in the absence of bone metastases. [3] Hypercalcemia of malignancy is strongly associated with a poor prognosis. The evidence is consistent across multiple cancer types, including urothelial carcinomas [4]. Median survival was 25 to 52 days for solid organ malignancies in PTHrP-mediated hypercalcemia [4]. Higher PTHrP levels are independently associated with worse survival, even after adjusting for calcium concentration [5].

Multiple large epidemiological studies consistently demonstrate that kidney stones are associated with urothelial carcinoma [6-8]. Specifically, staghorn calculi carry the highest association among the stone types [9]. The proposed mechanisms include chronic mechanical irritation, oxidative stress, DNA damage, and prolonged urothelial exposure to carcinogens due to obstruction [6]. This relationship may not be purely unidirectional. There is also evidence that tumor-related obstruction may alter urinary flow dynamics and lead to stasis, predisposing to urolithiasis [10]. 

In either case, metabolic derangement is likely to have further contributed to the development of the staghorn calculus. In states of chronic hypercalcemia, renal tubular re-absorptive capacity is overwhelmed and urinary calcium excretion increases. The resultant hypercalciuria is a major risk factor for calcium-based stones, regardless of underlying cause [11]. While staghorn calculi were historically considered struvite stones related to infection, more recent studies have shown that majority of staghorn calculi are in fact metabolic [12]. Of these, the most common stone composition is calcium phosphate [12]. This underscores the importance of metabolic evaluation for an underlying systemic cause, in the absence of readily available stone analysis, even in the setting of acute infection.

Often, as in this case, staghorn calculi are associated with significant morbidity [13]. This can be due to progressive renal deterioration, recurrent or severe urinary tract infections, and complications such as XGP and pyonephrosis [13]. XGP is characterized by suppurative inflammation and its replacement with yellowish granulomatous tissue leading to renal parenchymal destruction [1]. XGP may diagnostically mimic urological malignancy on both clinical presentation and imaging. Patients with either condition may present with weight loss, anemia, leukemoid reaction, and a renal mass [1,2]. Typical radiological findings on CT include the "bear paw sign," as seen in Figure 2, which describes an enlarged kidney with multiple low-attenuation rounded spaces representing dilated calyces, surrounded by thin renal parenchyma with higher contrast enhancement - mimicking the appearance of the dark toe pads on a bear’s paw [1,14]. On PET-CT, the imaging findings of XGP are non-specific as high FDG-avidity is seen in both inflammatory and neoplastic processes [1]. As such, tissue biopsy may be necessary for definitive diagnosis in select cases. Given the diagnostic mimicry and the possibility of co-existence of XGP with urological tumors [15-17], it is prudent to maintain a high index of suspicion to avoid a missed or incorrect diagnosis.

In line with this patient's preferences of care and in light of her oncological prognosis of less than one year, a tissue biopsy or a nephrectomy of the left kidney were not pursued. The diagnosis of XGP was hence presumed based on radiological features, and the co-existence of metastatic urothelial carcinoma was made considering clinical, radiological, and histopathological features. Specifically, the co-positivity of immunohistochemistry markers GATA3 and p40 is highly indicative of urothelial carcinoma metastases [18]. This differentiates it from the less likely diagnosis of SCC, which is p40 positive and GATA3 negative [19]. Nonetheless, a systematic evaluation was made for any clinical and radiological features suggestive of SCC, relating to the aerodigestive tract, lungs, or gynaecological system, for which there were none, further supporting the diagnosis of metastatic urothelial carcinoma.

## Conclusions

This case illustrates the complex interplay between severe renal infection and underlying malignancy, highlighting the diagnostic challenges posed by overlapping clinical and radiological features. XGP can closely mimic, and rarely co-exist with, urothelial carcinoma, particularly in the setting of chronic obstruction and nephrolithiasis. The presence of PTH-independent hypercalcemia, especially with elevated PTHrP, served as a critical clue prompting further evaluation for occult malignancy in this patient. Timely recognition and management of hypercalcemia and sepsis were essential in stabilizing the patient, while advanced imaging and tissue diagnosis ultimately established the presence of metastatic urothelial carcinoma.

Within the limitations of a single case report, the case serves as a reminder that diagnostic vigilance is crucial - specifically, active recognition that multiple pathologies can co-exist. A multidisciplinary approach remains pivotal in guiding both diagnostic evaluation and individualized management in such complex presentations.
